# Monthly Summary

**Published:** 1869-11

**Authors:** 


### MONTHLY SUMMARY.

---

*To Cool Water.*—In order to cool water by evaporation, it has long been the practice in warm countries to wrap the pitcher or other vessel containing the water with a wet cloth, the evaporation from which served to reduce the temperature of the vessel and consequently of its contents. An English manufacturing firm have applied the same principle in a portable refrigerator. The inner vessel for holding the liquid or substance to be cooled is surrounded by an an outer one containing water, and closed at the top by a layer of porous textile material. This latter draws up the water by capillary attraction, and the water, evaporating from the upper surface, produces the requisite reduction of temperature.—*Druggist's Circular.*

---

*A New Substitute for Phosphorus in Lucifer Matches.*—Dr. H. Fleck (*London Chemical News*) has substituted sodium for phosphorus in the manufacture of lucifer matches. His idea has been taken up by some of the largest manufacturers of lucifer and fusee matches. There is not the slightest danger in the transport. —*Med. Record.*

